# Endobronchial abnormalities and bronchoalveolar lavage findings in children with severe pneumonia and *Staphylococcus aureus*-positive transnasally suctioned airway secretion cultures: a retrospective study

**DOI:** 10.3389/fped.2026.1959006

**Published:** 2026-09-23

**Authors:** Haiqin Zhong, Zeyu Zeng, Haoxiang Gu, Lang Yuan, Xiaoyan Dong

**Affiliations:** Department of Respiratory Medicine, Shanghai Children’s Hospital, School of Medicine, Shanghai Jiao Tong University, Shanghai, China

**Keywords:** bronchoalveolar lavage fluid, bronchoscopy, child, severe pneumonia, *Staphylococcus aureus*

## Abstract

**Background:**

We aimed to describe endobronchial abnormalities and bronchoalveolar lavage fluid (BALF) findings in children with severe pneumonia and *Staphylococcus aureus*-positive transnasally suctioned airway secretion cultures.

**Methods:**

This retrospective study included 68 children hospitalized between January 2023 and December 2025. Bronchoscopic and BALF findings were evaluated in 34 children. Exploratory comparisons used nonparametric or categorical tests, with false-discovery-rate correction for 11 BALF comparisons.

**Results:**

Forty-eight children (70.6%) had at least one additional respiratory pathogen. Respiratory support was required in 34, intensive care admission in 17, and invasive ventilation in 11. The bronchoscopy subgroup was older and required less respiratory support, reflecting clinical selection. Retained secretions or mucus plugs were identified in 24 of 34 children (70.6%), bronchial casts in 10 (29.4%), and mucosal necrosis or necrosis-like changes in 6 (17.6%). *Staphylococcus aureus* was recovered from BALF culture in 4 of 34 children (11.8%). The median BALF neutrophil proportion was 70.0%. None of the 11 BALF comparisons remained significant after false-discovery-rate correction.

**Conclusion:**

A spectrum of endobronchial abnormalities was documented in this clinically selected subgroup, and BALF showed predominantly neutrophilic inflammation. The findings do not establish *Staphylococcus aureus*-specific causation or a therapeutic benefit of bronchoscopy.

## Introduction

*Staphylococcus aureus* can cause severe community-acquired pneumonia in children. The disease may progress rapidly and lead to bilateral pulmonary involvement, necrotizing pneumonia, lung abscess, pleural effusion, empyema, respiratory failure, and the need for intensive care or mechanical ventilation (1–3). Several virulence mechanisms, including cytotoxin production and induction of an intense neutrophilic response, contribute to epithelial injury, tissue necrosis, and suppurative inflammation (2, 3). Viral–bacterial interactions may further influence the severity and pathogenesis of respiratory infections (4, 5). Early recognition and appropriate antimicrobial therapy are therefore important in suspected severe *S. aureus* pneumonia.

Prior literature has examined the molecular epidemiology of community-acquired methicillin-resistant *S. aureus* (6), antimicrobial treatment of serious pediatric *S. aureus* infections (7), and resistance surveillance in pediatric intensive care (8). However, conducting-airway manifestations in this clinical setting are less well characterized. Retained purulent secretions, mucus plugs, and necrotic material may obstruct segmental or lobar bronchi and contribute to atelectasis (9, 10). Bronchoscopy permits direct visualization of these abnormalities and, when clinically indicated, removal of obstructive material (9, 10). A previous small pediatric series reported purulent secretions and erosive or necrosis-like mucosal injury (10), but the frequency and spectrum of mucus plugging, bronchial casts, necrosis-like mucosal injury, and associated BALF inflammation in children with severe pneumonia and *S. aureus*-positive airway secretion cultures remain uncertain.

Microbiological attribution is particularly challenging in pediatric pneumonia because hypopharyngeal airway secretion specimens obtained by transnasal suction are nonsterile, and additional respiratory pathogens may be detected concurrently. The qualifying specimens in this study were not anterior-nares swabs; transnasal suction refers to the route used to introduce the suction catheter. Recovery of *S. aureus* from these specimens should therefore be interpreted together with the clinical and radiological findings. The study was not designed to establish that *S. aureus* caused the pneumonia or that the observed airway findings were *S. aureus*-specific. Rather, we aimed to characterize the clinical, microbiological, radiological, bronchoscopic, and BALF features of a clinically defined cohort of children with severe pneumonia and *S. aureus*-positive airway secretion cultures, with particular emphasis on the frequency and spectrum of endobronchial findings among children who underwent bronchoscopy.

## Methods

### Study design and participants

This single-center retrospective observational study was conducted at Shanghai Children's Hospital, a tertiary pediatric referral center. The electronic medical record system was searched for community-acquired pneumonia (CAP)-related hospitalizations between January 2023 and December 2025 using prespecified diagnostic categories for pneumonia. Because the search categories could overlap, duplicate records were removed using the unique hospitalization identifier. All patients meeting the eligibility criteria during the study period were included. Because of the retrospective and primarily descriptive design, no *a priori* sample size calculation was performed. This study was reported in accordance with the Strengthening the Reporting of Observational Studies in Epidemiology (STROBE) statement.

Eligible children were aged 1 month to <18 years and were retrospectively classified as having severe CAP according to the 2024 Chinese guideline (11). In addition, *S. aureus* had to be recovered from an airway secretion specimen obtained by transnasal suction within 24 h of admission, antimicrobial susceptibility results had to be available, and sufficient clinical data had to be available for assessment of the main study variables. Clinical and radiological findings were required to be compatible with bacterial pneumonia. The clinical relevance of the *S. aureus* isolate was assessed independently by two pediatric respiratory physicians after integrated review of the clinical, radiological, and microbiological findings, including concurrently detected respiratory pathogens. An isolate was considered clinically relevant for cohort inclusion only when both physicians agreed. This adjudication was used solely to determine cohort eligibility and was not considered proof that *S. aureus* was the lower-respiratory-tract pathogen.

According to the 2024 Chinese guideline, severe CAP was defined by the presence of at least one of the following: poor general condition, refusal of feeding or signs of dehydration, altered consciousness, marked tachypnea, cyanosis, respiratory distress, oxygen saturation ≤92% on room air, multilobar involvement or involvement of at least two-thirds of a single lobe, pleural effusion, or an extrapulmonary complication. Supplemental oxygen was not an obligatory criterion under this definition because severity could be established by clinical, oxygenation, radiological, pleural, or extrapulmonary features. Individual children could meet more than one severity criterion; therefore, criterion-specific counts were not mutually exclusive.

Children were excluded if they did not meet the 2024 Chinese guideline definition of severe CAP; had pre-existing asthma, cystic fibrosis, obstructive sleep apnea, congenital heart disease, or documented cardiac dysfunction or heart failure; had an *S. aureus* isolate judged to be more consistent with colonization or contamination; or lacked key clinical data required for the study. Because the airway secretion specimens were obtained from a nonsterile site and additional respiratory pathogens were frequently detected, the study population is described as children with severe pneumonia and *S. aureus*-positive airway secretion cultures rather than as having pneumonia caused solely by *S. aureus*.

The study was approved by the Ethics Committee of Shanghai Children's Hospital on 9 May 2023 (approval no. 2023R051-F01). The Ethics Committee waived the requirement for written informed consent from participants' legal guardians because of the retrospective design and the use of routinely collected, de-identified clinical data.

### Data collection and definitions

Demographic, clinical, laboratory, microbiological, radiological, bronchoscopic, and bronchoalveolar lavage fluid (BALF) data were extracted from the electronic medical records and verified for accuracy. Peripheral white blood cell count, neutrophil proportion, C-reactive protein, procalcitonin, and lactate dehydrogenase were defined as the first available measurements obtained within 24 h after admission. The main descriptive outcome was the frequency and spectrum of endobronchial abnormalities among children who underwent bronchoscopy. Additional variables of interest included BALF cellular and cytokine profiles, radiological findings and complications, respiratory support requirements, pediatric intensive care unit (PICU) admission, and in-hospital outcomes.

Respiratory support was categorized according to the highest level received during hospitalization as low-flow oxygen delivered by nasal cannula or face mask, high-flow nasal cannula oxygen therapy, noninvasive ventilation, or invasive mechanical ventilation. Because the fraction of inspired oxygen and arterial blood gas measurements were not consistently available at standardized time points, oxygenation index and oxygen saturation index could not be calculated reliably for all participants. The interval from antimicrobial initiation to BALF sampling was calculated from the earliest reliably documented antimicrobial start date; when treatment had begun before admission, the documented pre-admission start date was used. This interval was available for all 34 children who underwent bronchoscopy.

Airway secretion specimens were obtained within 24 h of admission by trained nurses using a sterile suction catheter introduced through the nasal cavity to the hypopharynx. The collected specimens were hypopharyngeal airway secretions rather than anterior-nares swabs. Only specimens accepted for routine bacterial culture by the hospital clinical microbiology laboratory were included. *Staphylococcus aureus* identification and antimicrobial susceptibility testing were performed using the VITEK 2 automated microbial identification and susceptibility testing system (bioMérieux, Marcy-l'Étoile, France). Results were interpreted according to the CLSI M100 standards in effect during the study period. Isolates with an oxacillin minimum inhibitory concentration (MIC) of ≤2 μg/mL were classified as methicillin-susceptible *S. aureus* (MSSA), whereas isolates with an MIC of ≥4 μg/mL were classified as methicillin-resistant *S. aureus* (MRSA). MSSA/MRSA classification throughout the study was based on the qualifying transnasally suctioned airway secretion isolate rather than on BALF culture.

Additional respiratory pathogen detection was defined as identification of at least one respiratory pathogen other than *S. aureus* during the same hospitalization by culture, antigen testing, or nucleic acid amplification testing. Because multiple pathogens could be detected in the same child, pathogen-specific counts were not mutually exclusive.

All radiological variables reported in this study were derived from chest computed tomography (CT). CT variables included bilateral lung involvement, consolidation, pleural effusion, necrotizing pneumonia, lung abscess, empyema, pneumothorax, pericardial effusion, and pulmonary embolism. Necrotizing pneumonia was defined as multiple low-attenuation or liquefactive areas, with or without cavitation, within pulmonary consolidation.

### Bronchoscopy and BALF assessment

Bronchoscopy was performed as part of clinical care according to clinical indications rather than a predefined research protocol; no procedure was undertaken solely for research sampling. Clinical indications generally included extensive or segmental consolidation, consolidation with atelectasis, or suspected airway obstruction based on radiological and clinical findings. No child was electively endotracheally intubated solely to facilitate bronchoscopy; endotracheal intubation and invasive mechanical ventilation were used only when clinically indicated for respiratory failure. Intravenous midazolam was used for procedural sedation. Flexible bronchoscopy was performed using an Olympus BF-P260F or BF-XP260F bronchoscope (Olympus, Tokyo, Japan), selected according to patient size and procedural requirements. In children who were not intubated at the time of bronchoscopy, the bronchoscope was introduced transnasally. In the two children who were intubated at the time of bronchoscopy, the procedure was performed through the existing endotracheal tube. The bronchopulmonary segment showing the predominant radiological abnormality was selected for lavage. After the bronchoscope was wedged into the target bronchus, sterile 0.9% saline warmed to 37 °C was instilled through the working channel in aliquots of 1 mL/kg, with a maximum of 20 mL per aliquot and a total instilled volume of 3–5 mL/kg. BALF was recovered using negative-pressure suction at 100–200 mmHg, adjusted to avoid bronchial collapse during aspiration. A BALF recovery rate of ≥40% was targeted, consistent with the Chinese clinical practice guideline for pediatric bronchoalveolar lavage (9). The recovered BALF was submitted for microbiological, cellular, and cytokine analyses.

Bronchoscopic reports and available images were reviewed independently by two pediatric respiratory physicians using three predefined descriptive categories: retained secretions or mucus plugs, mucosal necrosis or necrosis-like changes, and bronchial casts. Retained secretions or mucus plugs were recorded when thick or tenacious secretions or mucus plugs were directly visualized and documented in the bronchoscopic report. Mucosal necrosis or necrosis-like changes were defined as visually apparent abnormalities judged on direct inspection to represent mucosal necrosis or a necrosis-like appearance; histopathological confirmation was not available. Bronchial casts were defined as organized, cohesive material conforming to the bronchial lumen or branching airway contour. A finding was recorded when both reviewers agreed; any initial disagreement was resolved by consensus. More than one category could be assigned to the same child. Because the retrospective source material did not permit reliable semiquantitative grading, no bronchoscopic severity score was retrospectively assigned. When obstructive secretions, mucus plugs, or casts were identified, bronchoscopic lavage and removal were performed as part of routine clinical care.

BALF total leukocyte counts were measured using an automated cell counter. Cell pellets were obtained by centrifugation, prepared as stained smears, and examined by light microscopy for differential cell counting. Inflammatory cytokines (IL-1β, IL-6, IL-8, IL-10, IL-17, IFN-*γ*, and TNF-α) were measured as part of routine clinical care in the hospital clinical laboratory using multiplex bead-based immunoassays. Cytokine results were retrospectively extracted from the electronic medical record. Cytokine measurements were available for 31 of the 34 children who underwent bronchoscopy (91.2%). Cytokine concentrations were summarized descriptively; comparisons according to methicillin-susceptibility phenotype were *post hoc* and exploratory.

### Statistical analysis

Statistical analyses were performed using SPSS version 23.0 (IBM Corp., Armonk, NY, USA). Continuous variables were presented as median (interquartile range), and categorical variables as number and percentage. Comparisons between children who did and did not undergo bronchoscopy, as well as BALF cellular and cytokine comparisons between the MSSA and MRSA groups, were exploratory. Continuous variables were compared using the Mann–Whitney U test, and categorical variables using the chi-square test or Fisher exact test, as appropriate. All tests were two-sided. Effect sizes were reported as r values (r = Z/√N, where N represents the total number of observations included in the corresponding comparison) for continuous variables and absolute risk differences for categorical variables. For the 11 BALF cellular and cytokine comparisons between the MSSA and MRSA groups, Benjamini–Hochberg false-discovery-rate correction was applied, and q values were reported alongside nominal *P* values. In *post hoc* exploratory analyses, Spearman rank correlations were used to assess associations between BALF neutrophil percentage or each of the seven cytokines and total hospital length of stay. Benjamini–Hochberg false-discovery-rate correction was applied across these eight correlation tests. Additional *post hoc* analyses compared seven cytokines by bronchial-cast and mucosal-necrosis status (14 Mann–Whitney tests), three bronchoscopic findings by methicillin susceptibility and additional-pathogen detection (six Fisher exact tests), and seven cytokines by additional-pathogen detection (seven Mann–Whitney tests). Benjamini–Hochberg correction was applied within each family. Missing data were not imputed; actual denominators were reported where applicable, and analyses were based on available cases. Multivariable analysis was not performed because of the limited sample size and the primarily descriptive nature of the study.

## Results

### Study flow

A total of 8,718 unique CAP hospitalizations were identified from the electronic medical record system during the study period. Among these, 291 records with an *S. aureus*-positive transnasally suctioned airway secretion culture were assessed for eligibility. A total of 223 records were excluded: 189 did not meet the criteria for severe pneumonia, 21 met a prespecified underlying-disease exclusion criterion, and 13 had a culture judged to represent colonization or contamination. The final cohort comprised 68 children, including 47 with MSSA and 21 with MRSA. Bronchoscopy with BALF sampling was performed in 34 children, whereas 34 did not undergo bronchoscopy. BALF cellular measurements and cultures were available for all 34 children undergoing bronchoscopy, and cytokine measurements were available for 31. *S. aureus* was recovered from BALF culture in 4 children. The participant-selection process is shown in Figure 1.

**Figure 1:**
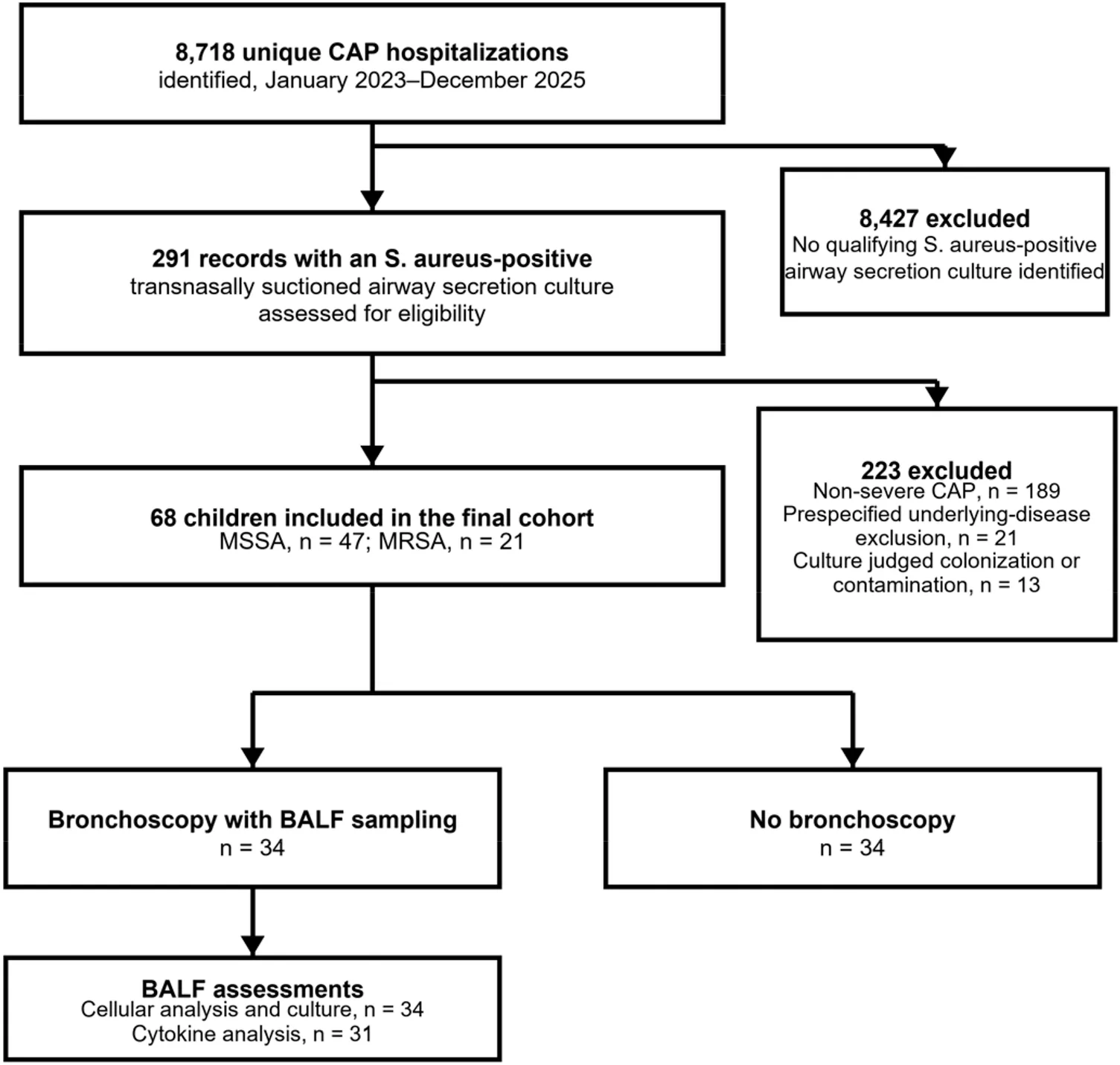
Flowchart of participant screening, eligibility assessment, and study inclusion. BALF, bronchoalveolar lavage fluid; CAP, community-acquired pneumonia; MRSA, methicillin-resistant *Staphylococcus aureus*; MSSA, methicillin-susceptible *Staphylococcus aureus*.

### Participant characteristics and laboratory findings

The cohort comprised 68 children, of whom 42 (61.8%) were boys. The median age was 5.1 years (IQR, 2.0–8.6), and the median length of hospital stay was 7 days (IQR, 5–10). Fever occurred in 61 children (89.7%), with a median duration of 5 days (IQR, 3–7) among those with fever. All children had cough, 37 (54.4%) had dyspnea, 2 (2.9%) had hemoptysis, and 1 (1.5%) reported chest pain.

An oxygen saturation ≤92% on room air was documented in 34 children (50.0%). Respiratory support was provided to 34 children (50.0%): low-flow oxygen by nasal cannula or face mask in 20, high-flow nasal cannula oxygen therapy in 2, noninvasive ventilation in 1, and invasive mechanical ventilation in 11. Seventeen children (25.0%) were admitted to the pediatric intensive care unit (PICU). The median peripheral white blood cell count was 9.66 × 10⁹/L (IQR, 6.54–13.24), the median neutrophil proportion was 70.1% (IQR, 56.0–80.6), the median C-reactive protein concentration was 14.6 mg/L (IQR, 5.0–39.1), and the median procalcitonin concentration was 0.14 ng/mL (IQR, 0.06–0.36). Clinical and laboratory characteristics according to bronchoscopy status are presented in Table 1.

**Table 1:** Clinical and laboratory characteristics according to bronchoscopy status.

Variable	Overall (*n* = 68)	Bronchoscopy (*n* = 34)	No bronchoscopy (*n* = 34)	Effect size	*P* value
Age, years, median (IQR)	5.1 (2.0, 8.6)	7.8 (5.2, 10.8)	2.5 (1.3, 4.6)	r = 0.565	<0.001
Male sex, n (%)	42 (61.8)	21 (61.8)	21 (61.8)	RD = 0.000	1.000
Hospital stay, days, median (IQR)	7.0 (5.0, 10.0)	7.0 (5.0, 10.0)	7.0 (5.0, 9.8)	r = −0.002	0.990
Fever duration among febrile children, days, median (IQR); *n* = 61	5.0 (3.0, 7.0)	5.0 (3.5, 7.0); *n* = 31	4.0 (3.0, 6.0); *n* = 30	r = 0.165	0.200
WBC, ×10⁹/L, median (IQR)	9.66 (6.54, 13.24)	8.9 (6.8, 12.1)	10.6 (6.5, 14.0)	r = −0.132	0.278
Neutrophils, %, median (IQR)	70.1 (56.0, 80.6)	65.7 (53.6, 79.1)	72.0 (60.2, 82.1)	r = −0.158	0.196
CRP, mg/L, median (IQR)	14.6 (5.0, 39.1)	15.5 (7.1, 67.7)	9.2 (5.0, 25.3)	r = 0.203	0.100
PCT, ng/mL, median (IQR)	0.14 (0.06, 0.36)	0.13 (0.07, 0.35)	0.14 (0.04, 0.36)	r = 0.100	0.410
LDH, U/L, median (IQR)	330 (290.8, 374.3)	317 (284, 367)	352 (292, 389)	r = −0.115	0.340
PICU admission, n (%)	17 (25.0)	4 (11.8)	13 (38.2)	RD = −0.265	0.023
Invasive mechanical ventilation, n (%)	11 (16.2)	2 (5.9)	9 (26.5)	RD = −0.206	0.045
Respiratory support, n (%)	34 (50.0)	10 (29.4)	24 (70.6)	RD = −0.412	0.001
Additional respiratory pathogen detected, n (%)	48 (70.6)	25 (73.5)	23 (67.6)	RD = 0.059	0.790
MRSA-positive airway secretion culture, n (%)	21 (30.9)	14 (41.2)	7 (20.6)	RD = 0.206	0.110

Values are n (%) or median (interquartile range). Continuous variables were compared using the Mann–Whitney U test; categorical variables were compared using the chi-square test or Fisher exact test, as appropriate. The WBC comparison used a tie- and continuity-corrected normal approximation. Effect sizes are effect-size r values (r = Z/√N) for continuous variables and absolute risk differences (RD; bronchoscopy minus no bronchoscopy) for categorical variables. MRSA classification was based on the qualifying transnasally suctioned airway secretion isolate. CRP, C-reactive protein; IQR, interquartile range; LDH, lactate dehydrogenase; MRSA, methicillin-resistant *Staphylococcus aureus*; PCT, procalcitonin; PICU, pediatric intensive care unit; WBC, white blood cell count.

### Microbiological findings

Among the 68 included children, MSSA was identified in 47 (69.1%) and MRSA in 21 (30.9%). No additional respiratory pathogen was detected in 20 children (29.4%), whereas at least one additional pathogen was identified in 48 (70.6%). The most frequently identified additional pathogens were rhinovirus (*n* = 17), *Mycoplasma pneumoniae* (*n* = 15), respiratory syncytial virus (*n* = 7), human bocavirus (*n* = 5), influenza A virus (*n* = 3), *Streptococcus pneumoniae* (*n* = 3), and *Haemophilus influenzae* (*n* = 3). Ten children (14.7%) had three or more additional respiratory pathogens identified.

### Radiological findings

All children underwent chest computed tomography, and all radiological findings reported below were based on CT. Bilateral lung involvement was present in 49 children (72.1%), consolidation in 46 (67.6%), pleural effusion in 9 (13.2%), and necrotizing pneumonia in 6 (8.8%). Lung abscess, empyema, pneumothorax, pericardial effusion, and pulmonary embolism were each documented in one child (1.5%). Representative computed tomography findings are shown in Supplementary Figure S1.

### Bronchoscopic and BALF findings

Bronchoscopy was performed in 34 of the 68 children (50.0%). Compared with children who did not undergo bronchoscopy, those who underwent the procedure were older (median, 7.8 vs. 2.5 years; *P* < 0.001) and less frequently required respiratory support (29.4% vs. 70.6%; *P* = 0.001), PICU admission (11.8% vs. 38.2%; *P* = 0.023), or invasive mechanical ventilation (5.9% vs. 26.5%; *P* = 0.045). These findings describe the subgroup of children who underwent bronchoscopy and should not be interpreted as prevalence estimates for the entire cohort.

Bronchoscopy with BALF sampling was performed a median of 3 days after admission (IQR, 2–4 days) and a median of 6 days after initiation of antimicrobial therapy (IQR, 3–8 days).

Retained secretions or mucus plugs were the most frequent bronchoscopic finding, occurring in 24 of 34 children (70.6%). Bronchial casts were directly visualized in 10 children (29.4%), and mucosal necrosis or necrosis-like changes were documented in 6 (17.6%). More than one bronchoscopic abnormality could be present in the same child. Representative findings are shown in Figure 2.

**Figure 2:**
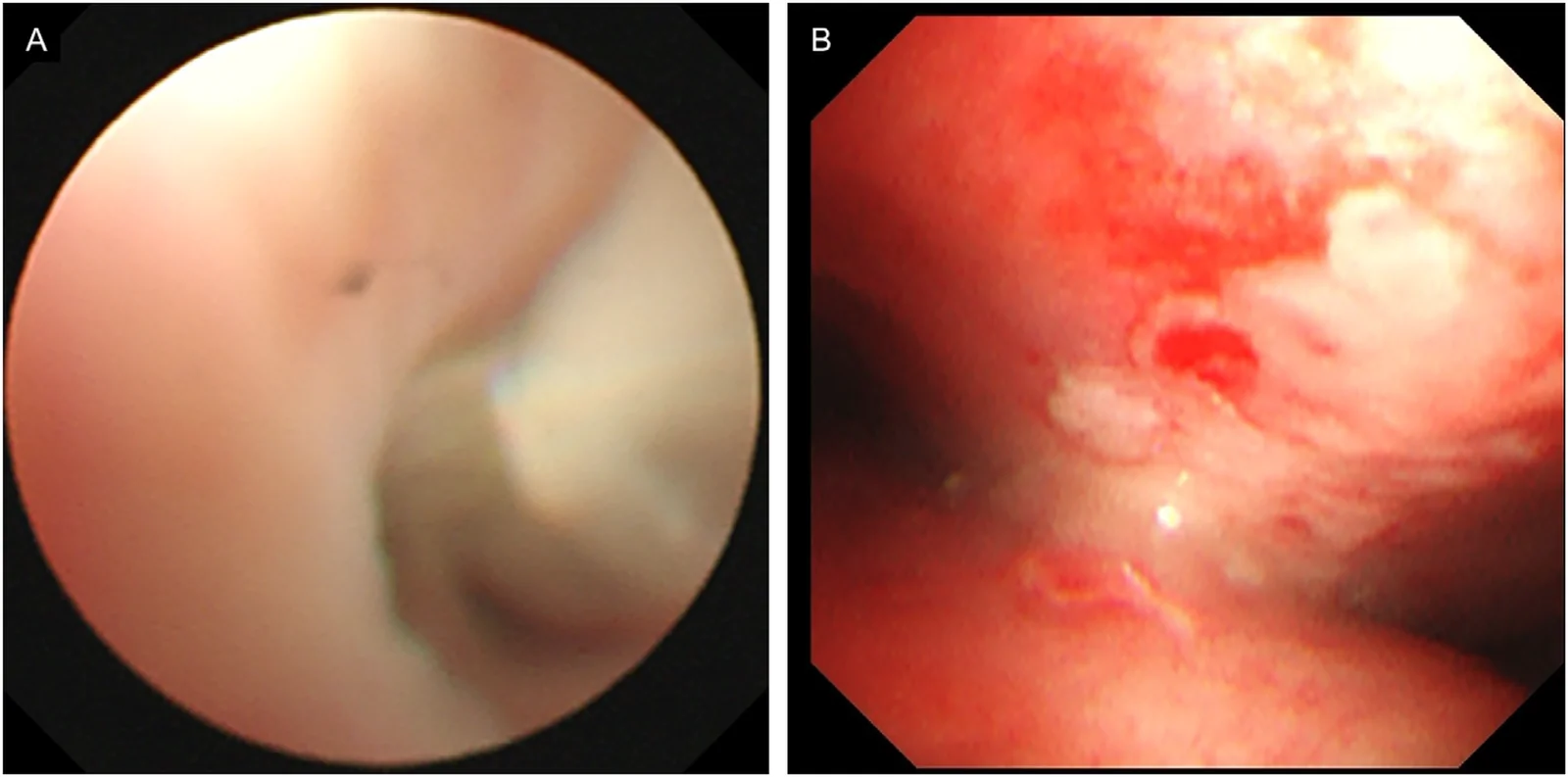
Representative bronchoscopic findings. **(A)** A white bronchial cast obstructing the bronchial lumen. **(B)** A necrosis-like bronchial mucosal change with adherent purulent secretions.

*Staphylococcus aureus* was recovered from BALF culture in 4 of the 34 children who underwent bronchoscopy (11.8%).

The median BALF total leukocyte count was 1,607 × 10⁶/L (IQR, 647.5–4,602.3). Neutrophils predominated, with a median proportion of 70.0% (IQR, 54.5–79.8); the median lymphocyte proportion was 15.5% (IQR, 8.3–19.0), and the median macrophage proportion was 16.0% (IQR, 11.0–21.0). Cytokine measurements were available for 31 children (91.2%). Overall BALF cellular and cytokine findings are presented in Table 2.

**Table 2:** Bronchoscopic findings and overall BALF characteristics among children undergoing bronchoscopy (*n* = 34).

Variable	Value
Bronchoscopic findings, n/N (%)	
Retained secretions or mucus plugs	24/34 (70.6)
Bronchial casts	10/34 (29.4)
Mucosal necrosis or necrosis-like changes	6/34 (17.6)
BALF microbiological finding	
BALF culture positive for *S. aureus*, n/N (%)	4/34 (11.8)
BALF cellular findings, median (IQR), *n* = 34	
Total leukocyte count, ×10⁶/L	1,607 (647.5, 4,602.3)
Neutrophils, %	70.0 (54.5, 79.8)
Lymphocytes, %	15.5 (8.3, 19.0)
Macrophages, %	16.0 (11.0, 21.0)
BALF cytokines, median (IQR), *n* = 31	
IL-1β, pg/mL	291.98 (56.36, 1,023.08)
IL-6, pg/mL	129.24 (30.15, 409.77)
IL-8, pg/mL	2,202.39 (897.41, 6,828.66)
IL-10, pg/mL	7.16 (2.44, 25.18)
IL-17, pg/mL	5.05 (2.84, 13.09)
IFN-*γ*, pg/mL	14.59 (2.44, 71.71)
TNF-α, pg/mL	13.88 (3.25, 52.05)

Values are median (interquartile range) unless otherwise indicated. Different bronchoscopic findings could coexist. BALF, bronchoalveolar lavage fluid; IFN, interferon; IL, interleukin; IQR, interquartile range; TNF, tumor necrosis factor.

Post hoc comparisons of BALF cellular and cytokine measurements between children with MSSA-positive and MRSA-positive airway secretion cultures are reported in Supplementary Table S1. Although IL-6, IL-1β, and TNF-α showed nominal between-group differences, none of the 11 comparisons remained significant after Benjamini–Hochberg false-discovery-rate correction (all q ≥ 0.076). In *post hoc* exploratory analyses, none of the correlations between BALF neutrophil percentage or the seven cytokines and hospital length of stay was significant after correction across eight tests (all q ≥ 0.186; Supplementary Table S2). Additional *post hoc* analyses found no FDR-adjusted cytokine differences by bronchial-cast or mucosal-necrosis status; IL-17 was nominally higher with necrosis-like changes (*P* = 0.008, q = 0.118). Bronchoscopic findings did not differ by methicillin susceptibility or additional-pathogen detection, and cytokines did not differ by additional-pathogen detection (Supplementary Tables S3–S5).

### Treatment and in-hospital outcomes

All children received antimicrobial therapy. During hospitalization, regimens included beta-lactam agents in 60 children, macrolide, minocycline, or moxifloxacin therapy with activity against atypical pathogens in 35, and linezolid or vancomycin in 9; these categories were not mutually exclusive. and 39 (57.4%) received methylprednisolone as part of individualized clinical management. Among the 31 children with cytokine measurements, 13 (41.9%) received methylprednisolone at some time during hospitalization. All 34 children undergoing bronchoscopy received bronchoalveolar lavage; obstructive secretions, mucus plugs, or casts were cleared when identified. Two children underwent chest tube drainage, and 1 subsequently required surgery for empyema. All 68 children improved clinically and were discharged alive; no in-hospital deaths occurred.

## Discussion

In this cohort of 68 children with severe pneumonia and *S. aureus*-positive airway secretion cultures, bronchoscopy revealed a spectrum of endobronchial abnormalities among the 34 children selected for the procedure: retained secretions or mucus plugs were identified in 70.6%, bronchial casts in 29.4%, and mucosal necrosis or necrosis-like changes in 17.6%. The bronchoscopy subgroup was older and less likely to require respiratory support, PICU admission, or invasive mechanical ventilation. This pattern may reflect indication-related selection, age-related procedural feasibility, and differences in clinical stability. No child was intubated solely to facilitate bronchoscopy. The reported bronchoscopic frequencies therefore apply to this clinically selected subgroup and not to all children meeting the Chinese definition of severe pneumonia.

Additional respiratory pathogens were detected in 70.6% of children. Because the qualifying specimens were nonsterile, *S. aureus* positivity cannot establish causation. *S. aureus* was recovered from BALF in only 4 of 34 children. The median 6-day antimicrobial interval referred to BALF sampling, which may have reduced culture yield; the qualifying hypopharyngeal specimen was obtained within 24 h of admission. These factors preclude *S. aureus*-specific attribution.

*Mycoplasma pneumoniae* was detected in 15 children, whereas *Streptococcus pneumoniae* and *Haemophilus influenzae* were each detected in 3. These pathogens may have contributed to disease severity or radiological and endobronchial findings, but their independent contributions could not be determined because the pathogen-specific subgroups were small and detections overlapped. Influenza A virus was co-detected in 3 children, whereas PVL status was not assessed. A published pediatric series of influenza A and PVL-positive *S. aureus* coinfection described conducting-airway involvement, including plastic bronchitis (12), but meaningful phenotype comparison was precluded by the limited number in our cohort.

Previous pediatric *S. aureus* studies have emphasized antimicrobial resistance, necrotizing pneumonia, empyema, and outcomes, with limited bronchoscopic characterization. The present study adds to a previous 12-patient series (10) by documenting mucus plugging, bronchial casts, and necrosis-like mucosal injury. These airway findings complement CT evidence of parenchymal and pleural abnormalities. Similar mucus plugs or casts occur in *Mycoplasma pneumoniae*, influenza, and other respiratory infections (12–16); without a direct comparator, whether the observed pattern differs from other severe pediatric pneumonias remains uncertain.

BALF neutrophil predominance was consistent with prior descriptions of neutrophil-dominant inflammation in bacterial pneumonia and *S. aureus* pulmonary infection (2, 17, 18), as well as the purulent bronchoscopic findings. Although IL-6, IL-1β, and TNF-α showed nominal differences between the MSSA and MRSA groups, none remained significant after false-discovery-rate correction; these findings should therefore be considered exploratory rather than evidence of a methicillin-susceptibility-specific inflammatory phenotype. Bronchial casts were observed in nearly one-third of children undergoing bronchoscopy. Cast formation has been associated with *M. pneumoniae*, influenza and other viral infections, and bacterial pneumonia (13–15). Neutrophil extracellular traps have been implicated in *M. pneumoniae*-associated cast formation (16). However, because cast composition and neutrophil extracellular traps were not assessed, the mechanism of cast formation in this cohort remains uncertain. Methylprednisolone was administered to 13 of the 31 children with cytokine measurements, but its variable timing and duration relative to BALF sampling introduced potential treatment-related confounding. The additional analyses identified no robust cytokine- or pathogen-defined bronchoscopic phenotype; the nominal IL-17 finding requires confirmation.

The principal clinical contribution of this study is the characterization of endobronchial involvement in children with severe pneumonia and *S. aureus*-positive hypopharyngeal secretion cultures. The combined CT, bronchoscopic, and BALF findings indicate that airway obstruction and mucosal injury may coexist with parenchymal disease, supporting consideration of airway involvement in selected children with persistent consolidation, atelectasis, or suspected bronchial obstruction. However, an *S. aureus*-positive hypopharyngeal culture does not prove lower-respiratory infection, and the therapeutic benefit of bronchoscopy remains unproven. BALF inflammatory measurements cannot currently guide pathogen attribution, antimicrobial selection, or risk stratification. Prospective studies with standardized lower-airway microbiology and longitudinal outcomes are needed.

Strengths include integration of clinical, microbiological, CT, bronchoscopic, and BALF data; universal CT; independent review of bronchoscopic findings; distinction between qualifying airway isolates and BALF cultures; and multiplicity adjustment for exploratory comparisons.

This single-center retrospective study has several limitations. The modest sample and Chinese severe-CAP definition limit generalizability, particularly to oxygenation-based critical-care populations. Nonsterile qualifying specimens, frequent pathogen co-detection, and limited BALF confirmation preclude pathogen-specific attribution. Clinically selected bronchoscopy introduces selection bias, so the observed airway findings cannot be generalized to the entire cohort. Despite standardized BAL procedures, variable sampling times, preceding antimicrobial exposure, and nonstandardized corticosteroid timing and duration may have influenced BALF measurements. Bronchoscopic assessment was qualitative, without a validated severity score. Exclusion of children with specified pre-existing cardiopulmonary conditions further limits applicability to those populations. The *post hoc* comparisons and correlations were limited by small sample sizes, residual confounding, and multiple testing. Prospective multicenter studies with standardized microbiological assessment, bronchoscopic evaluation, severity measures, and longitudinal outcomes are needed.

## Conclusion

Among children with severe pneumonia and *S. aureus*-positive airway secretion cultures who were selected for bronchoscopy, a spectrum of endobronchial abnormalities was documented, including retained secretions or mucus plugs, bronchial casts, and mucosal necrosis or necrosis-like changes. BALF showed predominantly neutrophilic inflammation. Because of microbiological uncertainty, retrospective clinical selection, and the absence of a disease-comparator or pathogen-negative group, these findings do not establish pathogen-specific causation or a therapeutic benefit of bronchoscopy.

## Data Availability

The datasets presented in this article are not readily available because of ethical and privacy restrictions. Requests to access the datasets should be directed to the corresponding author.
