# Supplementary material for: Endobronchial abnormalities and bronchoalveolar lavage findings in children with severe pneumonia and Staphylococcus aureus-positive transnasally suctioned airway secretion cultures: a retrospective study
Source: Front Pediatr. 2026 Sep 23;14:1959006. doi: 10.3389/fped.2026.1959006 (PMC13642759; doi:10.3389/fped.2026.1959006)
Supplement: Supplementary file 1 [file Supplementaryfile1.docx]

Supplementary Table S1. Exploratory BALF cellular and cytokine comparisons between the MSSA and MRSA groups

| **Variable** | **MSSA (cellular n = 20; cytokines n = 18)** | **MRSA (cellular n = 14; cytokines n = 13)** | **Effect size, r** | **P** | **BH-FDR q** |
| --- | --- | --- | --- | --- | --- |
| Total leukocyte count, ×10⁶/L | 2,834.50 (1,090.00–5,177.00) | 945.00 (501.00–1,672.00) | 0.282 | 0.100 | 0.227 |
| Neutrophils, % | 73.00 (65.00–84.00) | 66.50 (51.00–78.00) | 0.207 | 0.227 | 0.312 |
| Lymphocytes, % | 12.50 (6.00–18.00) | 17.00 (14.00–20.00) | −0.226 | 0.189 | 0.296 |
| Macrophages, % | 13.50 (11.00–19.00) | 18.50 (11.00–26.00) | −0.096 | 0.575 | 0.632 |
| IL-6, pg/mL | 229.68 (68.71–493.40) | 61.05 (13.14–131.96) | 0.406 | 0.024 | 0.087 |
| IL-1β, pg/mL | 698.40 (236.33–2,539.84) | 70.24 (12.63–459.51) | 0.442 | 0.014 | 0.076 |
| IL-8, pg/mL | 2,602.42 (882.07–7,500.00) | 1,461.78 (912.74–2,601.49) | 0.169 | 0.346 | 0.423 |
| TNF-α, pg/mL | 18.64 (9.61–98.71) | 2.70 (2.44–19.28) | 0.465 | 0.010 | 0.076 |
| IL-10, pg/mL | 9.12 (3.17–29.21) | 3.90 (2.44–9.51) | 0.293 | 0.103 | 0.227 |
| IL-17, pg/mL | 4.79 (3.79–14.67) | 7.17 (2.81–10.89) | 0.069 | 0.703 | 0.703 |
| IFN-γ, pg/mL | 27.05 (2.71–84.21) | 2.69 (2.44–18.69) | 0.253 | 0.160 | 0.293 |

Values are median (interquartile range). BALF cellular measurements were available for 20 children with MSSA and 14 with MRSA; cytokine measurements were available for 18 children with MSSA and 13 with MRSA. Comparisons used available cases, and missing values were not imputed. Comparisons used the Mann–Whitney U test. Effect sizes are effect-size r values (r = Z/√N, where N is the number analyzed for that comparison), with positive values indicating higher values in the MSSA group. Nominal P values and Benjamini–Hochberg false-discovery-rate-adjusted q values are reported. BALF, bronchoalveolar lavage fluid; IFN, interferon; IL, interleukin; IQR, interquartile range; MRSA, methicillin-resistant *Staphylococcus aureus*; MSSA, methicillin-susceptible *Staphylococcus aureus*; TNF, tumor necrosis factor.

Supplementary Table S2. Post hoc correlations of BALF inflammatory measurements with hospital length of stay

| **BALF variable** | **n** | **Spearman ρ** | **P value** | **BH-FDR q** |
| --- | --- | --- | --- | --- |
| Neutrophils, % | 34 | −0.381 | 0.026 | 0.186 |
| IL-6 | 31 | 0.053 | 0.776 | 0.776 |
| IL-1β | 31 | −0.307 | 0.093 | 0.186 |
| IL-8 | 31 | 0.178 | 0.339 | 0.387 |
| IL-10 | 31 | −0.307 | 0.093 | 0.186 |
| IL-17 | 31 | 0.259 | 0.160 | 0.213 |
| IFN-γ | 31 | −0.268 | 0.145 | 0.213 |
| TNF-α | 31 | −0.313 | 0.087 | 0.186 |

P values are two-sided. Benjamini–Hochberg false-discovery-rate correction was applied across the eight correlations. BALF, bronchoalveolar lavage fluid; BH-FDR, Benjamini–Hochberg false-discovery rate; IFN, interferon; IL, interleukin; TNF, tumor necrosis factor.

Supplementary Table S3. Post hoc BALF cytokine comparisons according to bronchial-cast and mucosal-necrosis status

| **Comparison** | **Finding present** | **Finding absent** | **Effect size, r** | **P** | **BH-FDR q** |
| --- | --- | --- | --- | --- | --- |
| Bronchial casts: IL-1β | 946.34 (103.73–1,046.15); n = 9 | 257.98 (36.64–733.63); n = 22 | 0.184 | 0.306 | 0.715 |
| Bronchial casts: IL-6 | 88.70 (62.28–232.99); n = 9 | 130.60 (26.00–414.16); n = 22 | 0.074 | 0.679 | 0.951 |
| Bronchial casts: IL-8 | 2,202.39 (715.91–6,868.75); n = 9 | 2,175.62 (1,044.76–5,947.36); n = 22 | -0.035 | 0.844 | 0.980 |
| Bronchial casts: IL-10 | 10.23 (3.17–29.21); n = 9 | 5.98 (2.44–11.70); n = 22 | 0.122 | 0.498 | 0.871 |
| Bronchial casts: IL-17 | 4.53 (4.41–13.40); n = 9 | 8.50 (2.53–12.31); n = 22 | 0.102 | 0.570 | 0.887 |
| Bronchial casts: IFN-γ | 42.24 (2.44–70.01); n = 9 | 8.04 (2.45–63.33); n = 22 | 0.047 | 0.793 | 0.980 |
| Bronchial casts: TNF-α | 14.77 (13.88–64.25); n = 9 | 8.69 (2.49–48.29); n = 22 | 0.278 | 0.122 | 0.491 |
| Mucosal necrosis or necrosis-like changes: IL-1β | 375.75 (282.72–640.32); n = 6 | 236.33 (42.48–1,142.74); n = 25 | -0.004 | 0.980 | 0.980 |
| Mucosal necrosis or necrosis-like changes: IL-6 | 265.29 (228.03–444.18); n = 6 | 75.20 (25.28–400.97); n = 25 | 0.265 | 0.140 | 0.491 |
| Mucosal necrosis or necrosis-like changes: IL-8 | 2,261.74 (1,353.59–2,531.39); n = 6 | 2,030.16 (719.58–6,868.75); n = 25 | 0.013 | 0.940 | 0.980 |
| Mucosal necrosis or necrosis-like changes: IL-10 | 37.31 (10.43–46.88); n = 6 | 5.21 (2.44–11.17); n = 25 | 0.339 | 0.059 | 0.415 |
| Mucosal necrosis or necrosis-like changes: IL-17 | 17.09 (10.96–107.74); n = 6 | 4.41 (2.44–10.88); n = 25 | 0.473 | 0.008 | 0.118 |
| Mucosal necrosis or necrosis-like changes: IFN-γ | 42.81 (5.76–269.22); n = 6 | 11.86 (2.44–51.39); n = 25 | 0.131 | 0.467 | 0.871 |
| Mucosal necrosis or necrosis-like changes: TNF-α | 36.40 (12.84–80.91); n = 6 | 11.70 (2.70–42.35); n = 25 | 0.184 | 0.304 | 0.715 |

Values are median (interquartile range). Comparisons used the Mann–Whitney U test. Positive effect-size r values indicate higher cytokine concentrations when the bronchoscopic finding was present. Benjamini–Hochberg correction was applied across all 14 comparisons. BALF, bronchoalveolar lavage fluid; BH-FDR, Benjamini–Hochberg false-discovery rate; IFN, interferon; IL, interleukin; TNF, tumor necrosis factor.

Supplementary Table S4. Post hoc comparisons of bronchoscopic findings according to methicillin-susceptibility phenotype and additional-pathogen detection

| **Comparison** | **First group, n/N (%)** | **Reference group, n/N (%)** | **Risk difference** | **P** | **BH-FDR q** |
| --- | --- | --- | --- | --- | --- |
| MRSA versus MSSA: Retained secretions or mucus plugs | 10/14 (71.4) | 14/20 (70.0) | 0.014 | 1.000 | 1.000 |
| MRSA versus MSSA: Bronchial casts | 2/14 (14.3) | 8/20 (40.0) | -0.257 | 0.141 | 0.848 |
| MRSA versus MSSA: Mucosal necrosis or necrosis-like changes | 2/14 (14.3) | 4/20 (20.0) | -0.057 | 1.000 | 1.000 |
| Any co-detected pathogen versus none: Retained secretions or mucus plugs | 19/25 (76.0) | 5/9 (55.6) | 0.204 | 0.395 | 1.000 |
| Any co-detected pathogen versus none: Bronchial casts | 7/25 (28.0) | 3/9 (33.3) | -0.053 | 1.000 | 1.000 |
| Any co-detected pathogen versus none: Mucosal necrosis or necrosis-like changes | 4/25 (16.0) | 2/9 (22.2) | -0.062 | 0.644 | 1.000 |

Comparisons used Fisher exact tests. Risk differences are first group minus reference group. Benjamini–Hochberg correction was applied across all six comparisons. BH-FDR, Benjamini–Hochberg false-discovery rate; MRSA, methicillin-resistant *Staphylococcus aureus*; MSSA, methicillin-susceptible *Staphylococcus aureus*.

Supplementary Table S5. Post hoc BALF cytokine comparisons according to additional respiratory-pathogen detection

| **Cytokine** | **Additional pathogen detected** | **No additional pathogen detected** | **Effect size, r** | **P** | **BH-FDR q** |
| --- | --- | --- | --- | --- | --- |
| IL-1β | 696.21 (88.28–1,495.76); n = 23 | 115.24 (25.98–324.60); n = 8 | 0.336 | 0.061 | 0.270 |
| IL-6 | 129.24 (46.59–479.10); n = 23 | 144.07 (12.39–244.18); n = 8 | 0.182 | 0.310 | 0.310 |
| IL-8 | 2,321.09 (1,050.65–8,510.61); n = 23 | 1,053.39 (629.71–3,052.47); n = 8 | 0.272 | 0.130 | 0.286 |
| IL-10 | 8.00 (3.54–26.30); n = 23 | 2.62 (2.44–13.88); n = 8 | 0.232 | 0.196 | 0.286 |
| IL-17 | 4.42 (2.83–10.00); n = 23 | 17.94 (10.20–47.69); n = 8 | -0.317 | 0.077 | 0.270 |
| IFN-γ | 15.61 (2.58–75.45); n = 23 | 2.80 (2.40–24.58); n = 8 | 0.228 | 0.205 | 0.286 |
| TNF-α | 13.88 (4.63–77.69); n = 23 | 12.04 (2.59–19.00); n = 8 | 0.191 | 0.288 | 0.310 |

Values are median (interquartile range). Comparisons used the Mann–Whitney U test. Positive effect-size r values indicate higher cytokine concentrations among children with additional-pathogen detection. Benjamini–Hochberg correction was applied across all seven comparisons. BALF, bronchoalveolar lavage fluid; BH-FDR, Benjamini–Hochberg false-discovery rate; IFN, interferon; IL, interleukin; TNF, tumor necrosis factor.

Supplementary Figure S1. Representative chest computed tomography (CT) findings. (A) Pulmonary consolidation. (B) Mediastinal-window CT showing necrotizing pneumonia. (C) Lung abscess. (D) Loculated pleural effusion in a child with empyema. The images are from four different children. Small red arrows indicate the illustrated abnormal regions.
