# Supplementary material for: Endobronchial abnormalities and bronchoalveolar lavage findings in children with severe pneumonia and Staphylococcus aureus-positive transnasally suctioned airway secretion cultures: a retrospective study
Source: Front Pediatr. 2026 Sep 23;14:1959006. doi: 10.3389/fped.2026.1959006 (PMC13642759; doi:10.3389/fped.2026.1959006)
Supplement: Supplementary file 2 [file Supplementaryfile2.docx]

STROBE Statement—Checklist of items that should be included in reports of ***cohort studies***

|  | Item No | Recommendation | Manuscript location / reporting details |
| --- | --- | --- | --- |
| **Title and abstract** | 1 | (*a*) Indicate the study’s design with a commonly used term in the title or the abstract | Title; Abstract, Methods (retrospective study). |
|  |  | (*b*) Provide in the abstract an informative and balanced summary of what was done and what was found | Abstract (Background, Methods, Results, and Conclusion). |
| Introduction | | |  |
| Background/rationale | 2 | Explain the scientific background and rationale for the investigation being reported | Introduction (clinical background, previous airway studies, and microbiological attribution). |
| Objectives | 3 | State specific objectives, including any prespecified hypotheses | Introduction, final paragraph (descriptive study objective); Methods, “Data collection and definitions” (main descriptive outcome). |
| Methods | | |  |
| Study design | 4 | Present key elements of study design early in the paper | Methods, “Study design and participants” (single-center retrospective observational design). |
| Setting | 5 | Describe the setting, locations, and relevant dates, including periods of recruitment, exposure, follow-up, and data collection | Methods, “Study design and participants” (Shanghai Children’s Hospital; January 2023–December 2025); Results, “Treatment and in-hospital outcomes” (in-hospital observation). |
| Participants | 6 | (*a*) Give the eligibility criteria, and the sources and methods of selection of participants. Describe methods of follow-up | Methods, “Study design and participants” (source population, eligibility, and exclusions); Results, “Study flow” and “Treatment and in-hospital outcomes”; Figure 1. Follow-up was limited to hospitalization. |
|  |  | (*b*) For matched studies, give matching criteria and number of exposed and unexposed | Not applicable—this was not a matched cohort study. |
| Variables | 7 | Clearly define all outcomes, exposures, predictors, potential confounders, and effect modifiers. Give diagnostic criteria, if applicable | Methods, “Study design and participants” (severe CAP and eligibility); “Data collection and definitions” (outcomes and measurements); “Bronchoscopy and BALF assessment” (bronchoscopic categories and BALF measurements). |
| Data sources/ measurement | 8* | For each variable of interest, give sources of data and details of methods of assessment (measurement). Describe comparability of assessment methods if there is more than one group | Methods, “Data collection and definitions” and “Bronchoscopy and BALF assessment” (record extraction; first available peripheral WBC count, neutrophil proportion, CRP, procalcitonin, and LDH obtained within 24 hours after admission; specimen source; assays; CT; and independent bronchoscopic image review). |
| Bias | 9 | Describe any efforts to address potential sources of bias | Methods, “Study design and participants” (clinical adjudication and exclusions) and “Bronchoscopy and BALF assessment” (independent review); Discussion (clinical selection, microbiological uncertainty, and limitations). |
| Study size | 10 | Explain how the study size was arrived at | Methods, “Study design and participants” (all eligible patients included; no a priori sample-size calculation); Results, “Study flow”; Figure 1. |
| Quantitative variables | 11 | Explain how quantitative variables were handled in the analyses. If applicable, describe which groupings were chosen and why | Methods, “Statistical analysis”; Tables 1–2 and Supplementary Tables S1–S2 (summaries, comparisons, effect sizes, and available-case denominators). |
| Statistical methods | 12 | (*a*) Describe all statistical methods, including those used to control for confounding | Methods, “Statistical analysis”; Table 1 footnote (WBC tie- and continuity-corrected normal approximation). No multivariable adjustment was performed. |
|  |  | (b) Describe any methods used to examine subgroups and interactions | Methods, “Statistical analysis” (exploratory bronchoscopy-status and MSSA–MRSA comparisons; post hoc BALF–hospital-stay correlations; BALF cytokine comparisons by bronchial-cast and mucosal-necrosis status; bronchoscopic findings by methicillin susceptibility and additional-pathogen detection; and cytokines by additional-pathogen detection). Benjamini–Hochberg correction was applied within the prespecified analysis families. Results are reported in Supplementary Tables S1–S5. No interaction analysis is reported. |
|  |  | (*c*) Explain how missing data were addressed | Methods, “Statistical analysis” (available cases; no imputation) and “Bronchoscopy and BALF assessment” (cytokine availability); Results, “Study flow” and “Bronchoscopic and BALF findings”; Table 1 (fever-duration denominators); Table 2; Supplementary Tables S1–S5. Bronchoscopic-category data were complete for all 34 children undergoing bronchoscopy; cytokine measurements were available for 31. |
|  |  | (*d*) If applicable, explain how loss to follow-up was addressed | Not applicable to post-discharge follow-up—this was an in-hospital retrospective study. Results, “Treatment and in-hospital outcomes”: all 68 children were discharged alive. |
|  |  | (*e*) Describe any sensitivity analyses | No formal sensitivity analysis was performed. Exploratory subgroup comparisons and correlations are reported under item 17. |
| Results | | |  |
| Participants | 13* | (a) Report numbers of individuals at each stage of study—eg numbers potentially eligible, examined for eligibility, confirmed eligible, included in the study, completing follow-up, and analysed | Results, “Study flow”; Figure 1: 8,718 CAP hospitalizations identified; 291 assessed; 223 excluded; 68 included; bronchoscopy/BALF in 34; cytokine measurements in 31. |
|  |  | (b) Give reasons for non-participation at each stage | Results, “Study flow”; Figure 1: non-severe pneumonia, n = 189; prespecified underlying-disease exclusion, n = 21; colonization/contamination, n = 13. |
|  |  | (c) Consider use of a flow diagram | Figure 1 (participant screening, eligibility assessment, inclusion, and BALF assessment availability). |
| Descriptive data | 14* | (a) Give characteristics of study participants (eg demographic, clinical, social) and information on exposures and potential confounders | Results, “Participant characteristics and laboratory findings” and “Microbiological findings”; Table 1 (overall cohort and bronchoscopy-status groups). |
|  |  | (b) Indicate number of participants with missing data for each variable of interest | Methods, “Statistical analysis”; Results, “Study flow” and “Bronchoscopic and BALF findings”; Table 1 (fever-duration denominators); Table 2; Supplementary Tables S1–S2 (measurement-specific denominators). A separate missing-data table for every variable is not provided. |
|  |  | (c) Summarise follow-up time (eg, average and total amount) | Results, “Participant characteristics and laboratory findings”; Table 1 (hospital stay: median 7 days, IQR 5–10). Observation was limited to hospitalization; total person-time is not reported. |
| Outcome data | 15* | Report numbers of outcome events or summary measures over time | Results, “Participant characteristics and laboratory findings,” “Bronchoscopic and BALF findings,” and “Treatment and in-hospital outcomes”; Tables 1–2. |
| Main results | 16 | (*a*) Give unadjusted estimates and, if applicable, confounder-adjusted estimates and their precision (eg, 95% confidence interval). Make clear which confounders were adjusted for and why they were included | Results; Tables 1–2; Supplementary Tables S1–S5 (descriptive summaries and unadjusted exploratory comparisons/correlations, with Benjamini–Hochberg false-discovery-rate correction for the specified analysis families). No confounder-adjusted estimates were calculated. Confidence intervals for effect estimates are not reported. |
|  |  | (*b*) Report category boundaries when continuous variables were categorized | Not applicable to the comparative analyses—continuous variables were not categorized for those analyses. Diagnostic thresholds used for eligibility and MSSA/MRSA classification are specified in Methods. |
|  |  | (*c*) If relevant, consider translating estimates of relative risk into absolute risk for a meaningful time period | Not applicable—no relative-risk estimates were calculated; Table 1 reports absolute risk differences. |
| Other analyses | 17 | Report other analyses done—eg analyses of subgroups and interactions, and sensitivity analyses | Methods, “Statistical analysis”; Results, “Bronchoscopic and BALF findings”; Table 1; Supplementary Table S1 (MSSA–MRSA comparisons) and Supplementary Table S2 (BALF–hospital-stay correlations). |
| Discussion | | |  |
| Key results | 18 | Summarise key results with reference to study objectives | Discussion, opening paragraph (principal endobronchial findings and clinical selection). |
| Limitations | 19 | Discuss limitations of the study, taking into account sources of potential bias or imprecision. Discuss both direction and magnitude of any potential bias | Discussion (clinical selection, microbiological uncertainty, small and overlapping pathogen-defined subgroups, multiple testing, qualitative bronchoscopic assessment, and final limitations paragraph). Variable corticosteroid timing and duration relative to BALF sampling are acknowledged as potential treatment-related confounding. Potential biases are discussed qualitatively; their magnitudes were not quantified. |
| Interpretation | 20 | Give a cautious overall interpretation of results considering objectives, limitations, multiplicity of analyses, results from similar studies, and other relevant evidence | Discussion; Conclusion (descriptive characterization of endobronchial involvement as the principal contribution; cautious interpretation considering microbiological uncertainty, clinical selection, multiplicity, corticosteroid exposure, and previous studies; and explicit recognition that the findings do not establish pathogen-specific causation, a validated risk-stratification approach, or therapeutic benefit). |
| Generalisability | 21 | Discuss the generalisability (external validity) of the study results | Discussion (clinical selection and final limitations paragraph); Conclusion (limits of applicability and causal interpretation). |
| Other information | | |  |
| Funding | 22 | Give the source of funding and the role of the funders for the present study and, if applicable, for the original study on which the present article is based | Funding (Scientific Research Project of the Shanghai Municipal Health Commission, No. 202340070; no funder role in study design, data collection, analysis, interpretation, manuscript preparation, or submission decision). |

Manuscript: Endobronchial abnormalities and bronchoalveolar lavage findings in children with severe pneumonia and Staphylococcus aureus-positive transnasally suctioned airway secretion cultures: a retrospective study

*Give information separately for exposed and unexposed groups.

**Note:** An Explanation and Elaboration article discusses each checklist item and gives methodological background and published examples of transparent reporting. The STROBE checklist is best used in conjunction with this article (freely available on the Web sites of PLoS Medicine at http://www.plosmedicine.org/, Annals of Internal Medicine at http://www.annals.org/, and Epidemiology at http://www.epidem.com/). Information on the STROBE Initiative is available at http://www.strobe-statement.org.
